# Upregulation of prefrontal metabotropic glutamate receptor 5 mediates neuropathic pain and negative mood symptoms after spinal nerve injury in rats

**DOI:** 10.1038/s41598-017-09991-8

**Published:** 2017-08-29

**Authors:** Geehoon Chung, Chae Young Kim, Yeong-Chan Yun, Sang Ho Yoon, Myoung-Hwan Kim, Yu Kyeong Kim, Sang Jeong Kim

**Affiliations:** 1 Department of Physiology, Seoul National University College of Medicine, Seoul, Korea; 2 Department of Brain and Cognitive Sciences, Seoul National University College of Natural Sciences, Seoul, Korea; 3 Department of Biomedical Sciences, Seoul National University College of Medicine, Seoul, Korea; 4 Department of Nuclear Medicine, Seoul National University College of Medicine, Seoul, Korea; 5 Neuroscience Research Institute, Seoul National University College of Medicine, Seoul, Korea

**Keywords:** Chronic pain, Depression, Prefrontal cortex, Neuropathic pain, Depression

## Abstract

Patients with chronic pain easily accompany the negative mood symptoms such as depression and anxiety, and these disturbances in turn affect the aversive perception of pain. However, the underlying mechanisms are largely unknown. We hypothesized that the alteration of metabotropic glutamate receptor 5 (mGluR5) in the brain region underlies such a comorbidity of aversive states. We scanned the brain of chronic neuropathic pain model rats using positron emission tomography (PET) technique with an mGluR5-selective radiotracer [11C] ABP688 and found various brain regions with higher or lower level of mGluR5 compared to control rats. Among the brain areas, a prominent upregulation of mGluR5 was shown in the prelimbic region (PrL) of the medial prefrontal cortex (mPFC) of chronic neuropathic pain animals. A pharmacological blockade of upregulated mGluR5 in the PrL ameliorated the negative symptoms including tactile hypersensitivity and depressive-like behavior, which relieved the subjects from the unpleasant state of chronic neuropathic pain condition. Conversely, lentiviral overexpression of the mGluR5 in the PrL of naïve rats successfully induced comorbid pain and negative moods. Our data provide deeper insight into the shared mechanism of pain perception and negative emotions, identifying a therapeutic target for the treatment of chronic pain and mood disorders.

## Introduction

Neuropathic pain persists even after the healing phase following an injury and patients suffer from symptoms including allodynia, hyperalgesia, and spontaneous pain. Central sensitization mechanisms of the pain system including the spinal cord and the brain are considered to be the main reason of such an unrelenting chronic pain.

As the pain goes chronic, supraspinal brain centers become crucial for how the patients perceive pain. Previous studies have demonstrated that the long-term pain arising from peripheral nerve injury induces maladaptive changes in the various cortical structures^1–6^ including the prefrontal cortex (PFC) which are also involved in the affective and emotional processing of the brain. Chronic neuropathic pain patients easily accompany the abnormal mental states such as depression or anxiety^7–9^, and these emotional mood symptoms in return affect the manifestation of the sensory pain symptoms^10–13^. This indicates that the shared supraspinal brain mechanisms play a critical role in the pathologically amplified negative perception in chronic pain condition.

One possible candidate is the alteration of metabotropic glutamate receptor 5 (mGluR5) in the brain. The mGluR5 is a G protein-coupled receptor which plays an important role in the modulation of neuronal excitability and is involved in the pathophysiology of various neurological and psychiatric disorders. The changes of mGluR5 expression in the brain regions and their functional impact have been reported from studies of chronic pain^14–16^ and negative mood disorders^17–19^. Both of pain processing and mental disorders are modulated by mGluR5 actions, implicating change of this receptor in the brain circuit as their common mechanisms. In the processing of pain and negative mood, the affective and cognitive interactions actively participate in the perception, and the maladaptive sensitization of this system in chronic pain condition amplifies negative appraisal and aversive sensations^20–22^. Our primary hypothesis is that the persistent unavoidable pain would trigger the alteration of mGluR5 in the brain area related to the negative appraisal such as the PFC, and this alteration, in turn, mediates pain facilitation and precipitates negative mood.

In this study, we sought to identify the brain alteration underlying the comorbidity of chronic pain and negative mood disorders. We used the mGluR5 as a target molecule, as this molecule is known to be involved in the pathophysiology of pain as well as negative moods. The common brain circuit was pursued by investigating the altered mGluR5 in the chronic pain state and was verified by effects of the local mGluR5 manipulation on the pain and mood behaviors. To assess the regional expression of mGluR5 among brains of chronic neuropathic pain rats and control rats, positron emission tomography (PET) technique was used with [11C] ABP688, a highly selective radiotracer of mGluR5. After this step, we went on further tests with pharmacological modulation and viral manipulation of mGluR5 in the identified brain area to investigate the specific role of the local mGluR5 alteration in the pain-related and mood-related behaviors.

## Results

### Altered mGluR5 level in the brain following nerve injury-induced neuropathic pain

**Figure 1:**
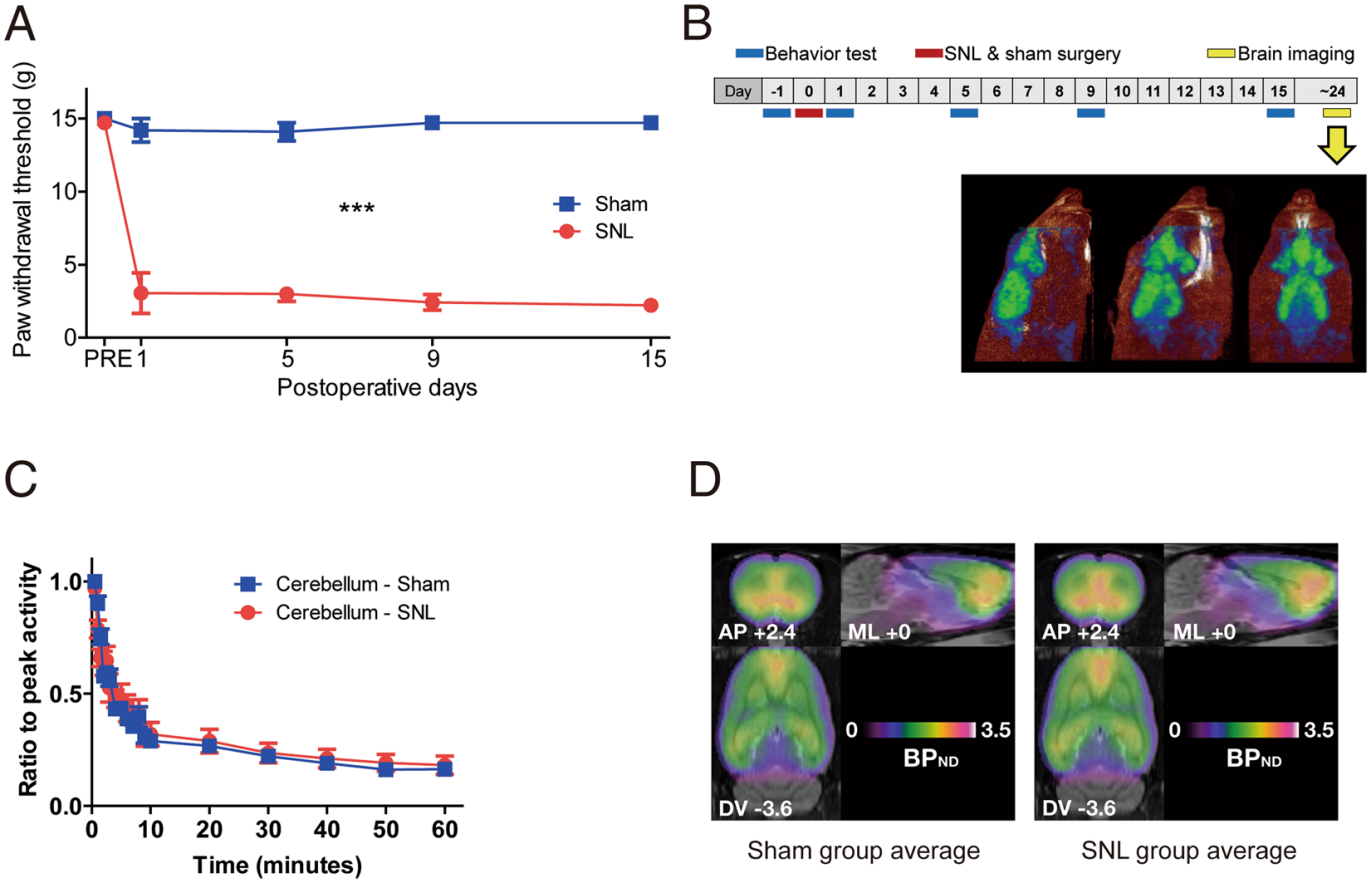
Experimental design and assessment of mGluR5 level in the brain. (**A**) After the surgery, SNL group animals showed reduced hindpaw withdrawal threshold compared to control group (n = 10 per each group, ***p < 0.001, Two-way repeated measures ANOVA with Bonferroni test). (**B**) Experimental design of [11C] ABP688 PET scan and sample image. PET scan was performed to animal 15–24 days after the SNL or sham surgery. (**C**) Time-activity curve of [11C] ABP688 in the cerebellum. The cerebellum was used as a reference region for the quantification of the mGluR5, as this region is devoid of the mGluR5. (**D**) Averaged [11C] ABP688 images from each group. The images without proportional scaling were used. The anterior-posterior (AP), mediolateral (ML), dorsoventral (DV) coordinates in the representative plane images are the distance from the bregma (mm).

**Figure 2:**
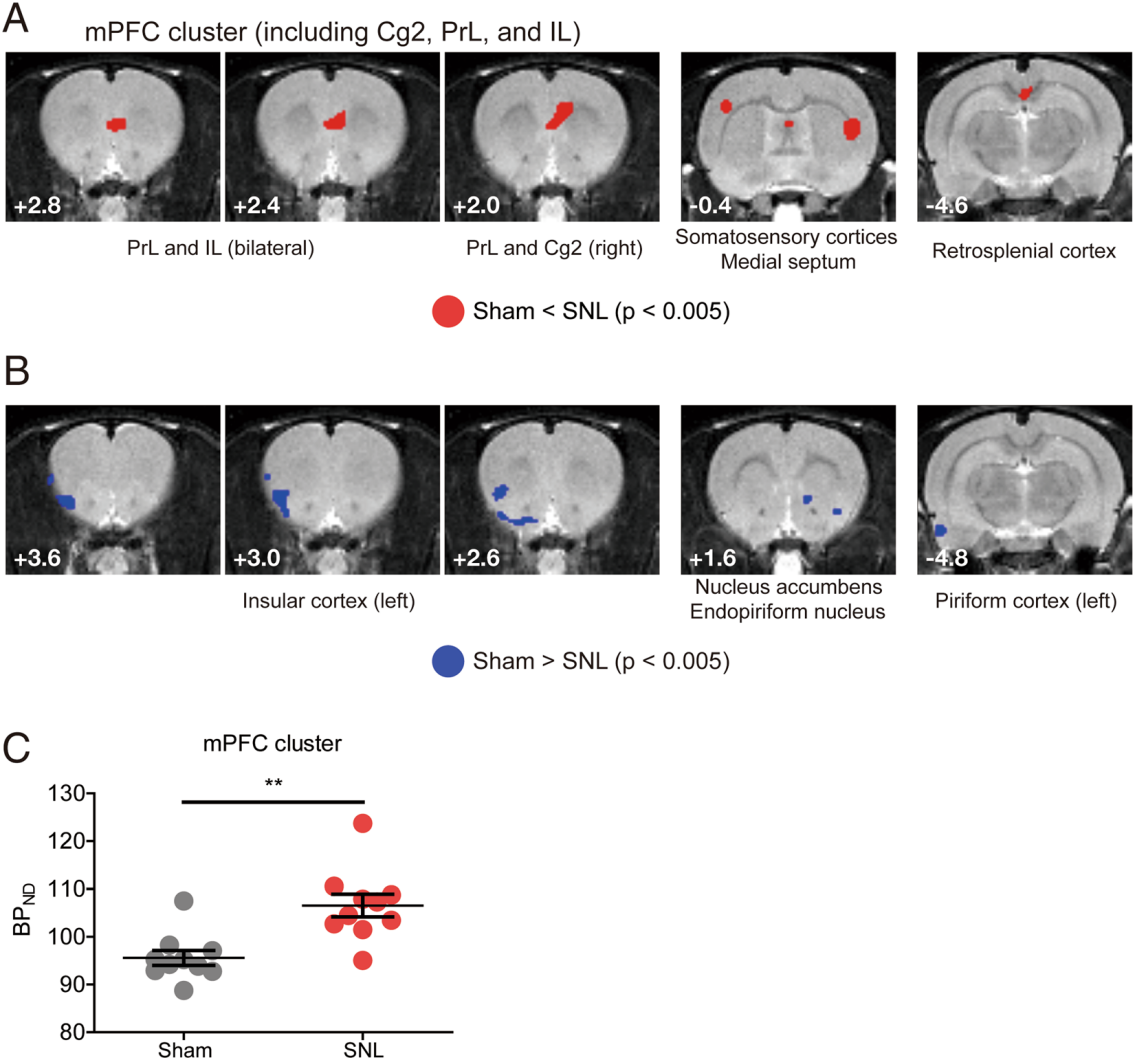
Comparison of mGluR5 level between SNL group and sham group animals. (**A**) Brain regions of which the mGluR5 level was higher in the SNL group compared to the sham control group. The PrL region of the SNL group showed the prominently higher level of mGluR5. (**B**) Brain regions with the lower mGluR5 level in the SNL group. (**C**) The mGluR5 level was higher in the mPFC cluster of SNL group animals (**p = 0.0011 with two sample t-test. Data of the cluster were extracted from proportionally scaled images).

Right L5 spinal nerve ligation (SNL) surgery-performed rats were used for chronic neuropathic pain group^23^ and sham surgery-performed rats were used for the control group. After surgery, paw withdrawal threshold was measured using von Frey test and SNL rats showed a consistent reduction of paw withdrawal threshold, which represents altered behavioral response due to nerve injury-induced mechanical allodynia (Fig. 1A). To identify the regional difference of brain mGluR5 in the chronic pain condition, an [11C] ABP688-PET scan was performed to SNL and sham group animal 15–24 days after surgery (Fig. 1B). The non-displaceable binding potential (BP_ND_) of [11C] ABP688 was calculated from each brain image with cerebellum as a reference region to assess the level of mGluR5 (Fig. 1C and D) and compared between groups on a voxel-by-voxel basis. The BP_ND_ of mGluR5 was found to be significantly different between SNL and sham rats in multiple pain-related brain regions (Fig. 2A and B, Table 1). SNL rats showed the significantly higher level of mGluR5 in the bilateral medial prefrontal cortex (mPFC) including caudal part prelimbic area (PrL), dysgranular zone of the primary somatosensory cortex (contralateral to nerve injury), the somatosensory cortices and caudate putamen (ipsilateral to nerve injury), retrosplenial cortex, and the medial septum (Fig. 2A). Conversely, the insular cortex (contralateral to nerve injury), the rostral pole of nucleus accumbens (ipsilateral to nerve injury), the endopiriform nucleus (ipsilateral to nerve injury), small areas of corpus callosum which contact to caudate putamen (ipsilateral to nerve injury), and piriform cortices and olfactory tubercle (contralateral to nerve injury) showed lower level of mGluR5 in the SNL group (Fig. 2B).

**Table 1:** Brain regions with significant differences between sham and SNL rats (two-sample t-test, p < 0.005, K_e_ > 20).

**Sham < SNL**							
Brain region	K_e_	T	Z	Peak p	Location (mm)		
					**ML**	**AP**	**DV**
Medial prefrontal cortex (Prelimbic and infralimbic cortices)	97	5.50	4.06	2.54E-06	0.2	2.6	−4.8
Primary somatosensory cortex (Dysgranular zone)	24	5.88	4.23	1.16E-05	−4.6	−0.2	−3.4
Primary, secondary somatosensory cortices and caudate putamen	37	4.42	3.52	0.0002	4.8	−0.4	−5.2
Retrosplenial cortex	25	6.70	4.56	2.45E-05	0.2	−4.6	−2.4
Medial septum	30	3.51	2.98	0.0014	0.4	−0.8	−4.8
**Brain region**	**K**_**e**_	**T**	**Z**	**Peak p**	**Location (mm)**		
					**ML**	**AP**	**DV**
Insular cortex and piriform cortex	102	7.64	4.89	4.98E-07	−3.2	3.6	−7.0
		4.73	3.69	0.0001	−3.4	3.0	−7.6
		4.60	3.62	0.0001	−3.6	2.6	−5.8
Insular cortex and primary somatosensory cortex	31	4.12	3.35	0.0004	−4.8	3.4	−4.8
Endopiriform nucleus	20	4.06	3.32	0.0005	3.8	1.4	−7.2
Nucleus accumbens	30	3.74	3.12	0.0009	1.6	1.8	−6.4
Piriform cortex	61	3.72	3.11	0.0009	−6.2	−4.8	−8.6
Corpus callosum and caudate putamen	36	3.71	3.11	0.0009	1.6	−0.4	−3.4
Corpus callosum and caudate putamen	76	3.65	3.07	0.0011	3.6	−2.0	−3.8

The peak voxel location is represented by the distance from the bregma (mm).

### Upregulation of mGluR5 in the mPFC of chronic neuropathic pain animals

We sought to identify a brain region with an altered mGluR5 level which affects to the amplified aversiveness in the chronic pain condition. Among the brain regions which showed prominent alteration of the mGluR5 level in neuropathic pain group, we focused on the mPFC cluster because of its suggested major roles in the mood disorders and affective perception^22,24–28^. According to previous studies, mGluR5 in the mPFC mediates depressive-like behavior^29^, and depressive and anxious symptoms following chronic pain are mediated by PrL subregions of the mPFC^30,31^. Brain images from our PET experiment showed that the mGluR5 level of the mPFC is increased in the neuropathic pain group (Fig. 2A). We conducted additional region-of-interest (ROI) analysis using BP_ND_ extracted from the mPFC cluster of each animal, which confirmed the significant increase of the mGluR5 level in the mPFC of SNL rats (Fig. 2C). We found that the significant cluster of the mPFC extracted from the analysis mainly consists of cingulate area 2 (Cg2) and caudal part PrL, and includes a small dorsal portion of the infralimbic cortex (IL). In the posterior part (anterior-posterior range (AP) + 1.4~2.4 mm from bregma) of the cluster, significant voxels were located in the Cg2 and the PrL, and the voxels of the deep layer ipsilateral to nerve injury were mainly included to the significant cluster. In the anterior part of the cluster (AP + 2.4~3.0 mm from bregma), significant voxels were located bilaterally in the ventral part of the PrL and dorsal part of the IL. The dorsoventral (DV) range of significant voxels in the mPFC cluster was −3.2~−5.2 mm from bregma.

### Pharmacological inactivation of mGluR5 in the caudal PrL ameliorates neuropathic mechanical allodynia

**Figure 3:**
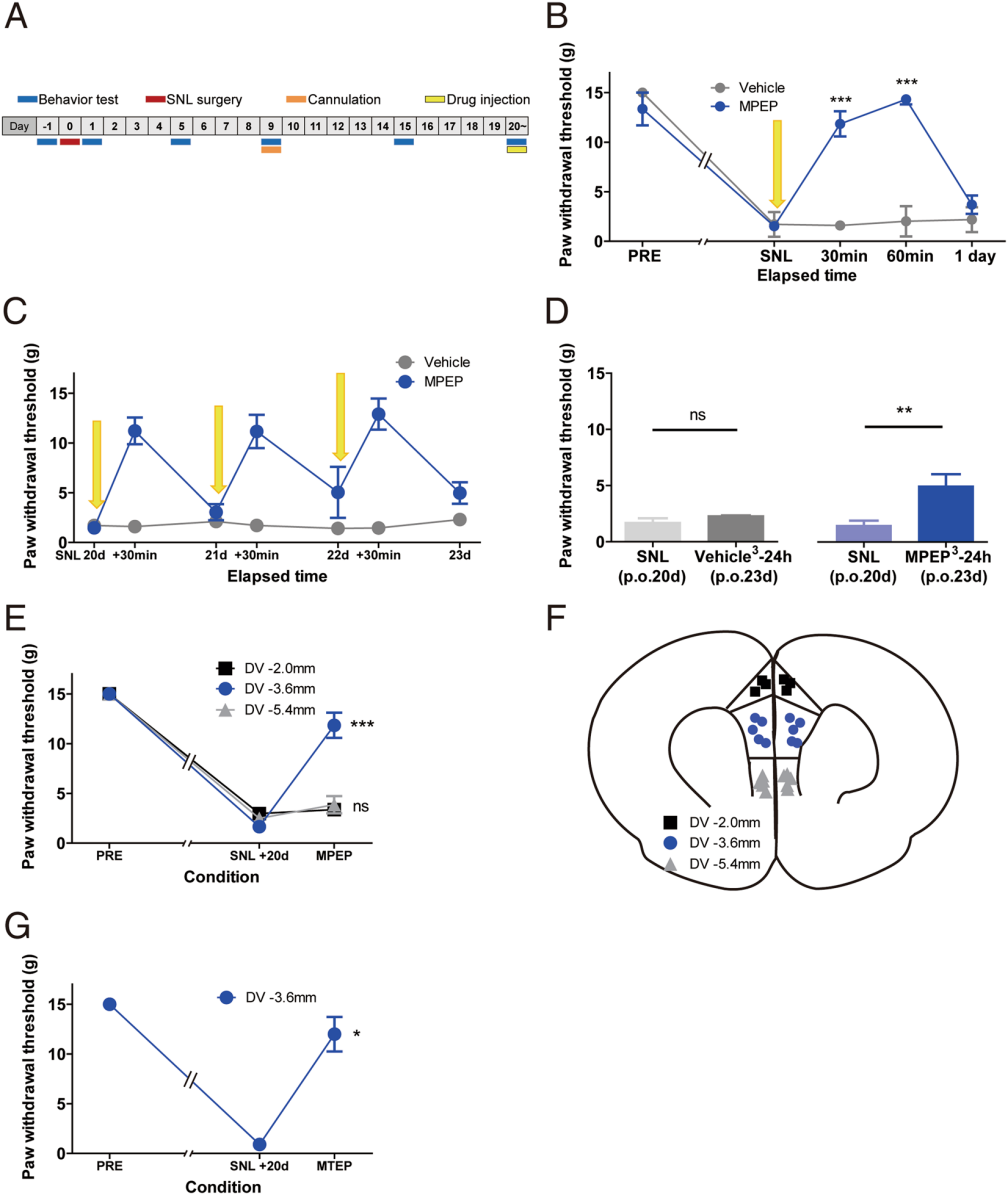
Blockade of mGluR5 within the PrL subregion of mPFC induces analgesic effect. (**A**) Experimental design. The MPEP injection and behavioral tests were performed at least 20 days after the SNL surgery. (**B**) Effect of MPEP injection into the mPFC on SNL-induced tactile hypersensitivity. PrL-MPEP treatment ameliorated SNL-induced mechanical allodynia symptom (n = 4~6 per each group, ***p < 0.001, Two-way repeated measures ANOVA with Bonferroni test). (**C**) Consecutive injection of MPEP into the PrL of SNL animals. The arrow indicates injection to the PrL. (**D**) The residual analgesic effect was shown 24 hours after the repeated PrL-MPEP treatment (n = 4~5 per each group, **p = 0.0066 with paired t-test, ns = not significant). (**E**) Off-target injection of MPEP failed to induce analgesic effect, which confirms that the effect of mPFC-MPEP injection on mechanical allodynia is PrL-specific (n = 3~7 per each group, ***p = 0.0003, paired t-test. (**F**) Confirmation of injection sites. (**G**) The injection of another mGluR5 antagonist MTEP into the PrL also successfully induced analgesic effect (n = 3, *p = 0.0273, paired t-test).

We wondered whether the increased mGluR5 in the mPFC affects the neuropathic pain behaviors. To identify its action on the peripheral nerve injury-induced tactile hypersensitivity, we injected mGluR5 antagonist 2-methyl-6-(phenylethynyl)pyridine (MPEP) into the bilateral mPFC (caudal part of the PrL) 20 days after SNL surgery via pre-implanted cannula and measured change of the paw withdrawal threshold (Fig. 3A). Administration of MPEP (PrL-MPEP treatment) evoked potent analgesic effect and significantly increased the paw withdrawal threshold. Consequently, mechanical allodynia was disappeared and paw withdrawal threshold was returned to the pre-SNL level within 30 minutes following MPEP injection (Fig. 3B).

Interestingly, the analgesic effect induced by PrL-MPEP treatment did not completely disappear with the time in a subset of SNL rats. Although mechanical allodynia was rebounded, paw withdrawal thresholds of SNL rats were slightly ameliorated at 24 hours after PrL-MPEP treatment compared to the pre-MPEP level. To further investigate this long-lasting effect, we repeated PrL-MPEP treatment for 3 consecutive days and measured paw withdrawal thresholds at 24 hours after the last injection (Fig. 3C). The paw withdrawal thresholds of SNL rats measured at 24 hours after the repeated PrL-MPEP treatment were significantly increased compared to the pre-MPEP level, which confirms the residual effect of the treatment (Fig. 3D).

To exclude the possibility that the effects we observed from MPEP injection were caused by the diffusion of the drug, we injected MPEP into the off-target sites and measured paw withdrawal threshold (Fig. 3E and F). The injection of the MPEP into the off-target sites failed to increase the paw withdrawal threshold of neuropathic pain animals. Administration of another mGluR5 antagonist MTEP also evoked similar analgesic effect (Fig. 3G). Taken together, these data clearly demonstrate that mGluR5 in the specific mPFC region plays a significant role in the pain processing. The effective mPFC subregion corresponds to the caudal part of the PrL.

### Blockade of increased mGluR5 in the PrL relieves neuropathic pain-induced depressive-like behavior

**Figure 4:**
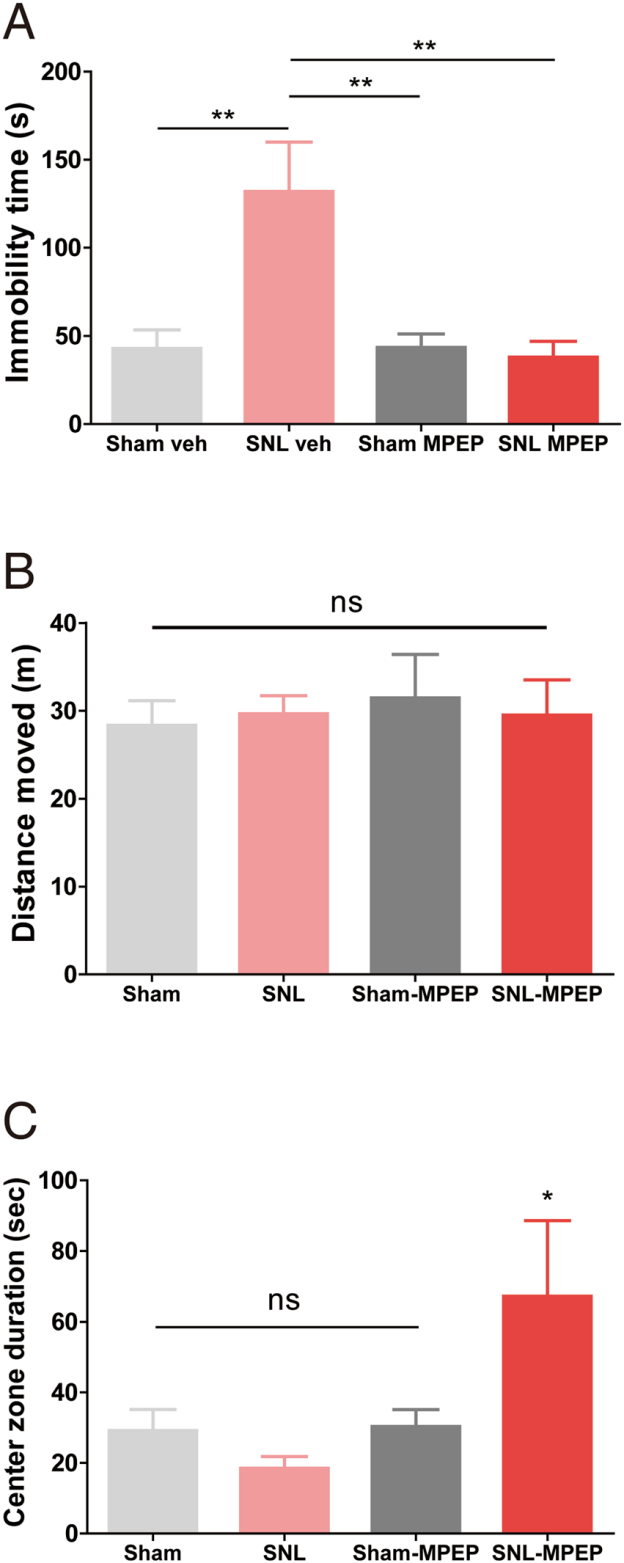
PrL-MPEP treatment induces antidepressive and anxiolytic effect only in SNL group. (**A**) SNL-induced increase of immobility time was rescued by PrL-MPEP treatment (n = 8–10 per each group, **p < 0.01, Bonferroni test in one-way ANOVA. (**B**) Neither SNL nor PrL-MPEP treatment altered total distance traveled in 5 min open field test. (**C**) Treatment of PrL-MPEP increased center zone duration only in SNL group (n = 4–7 per each group, *p < 0.05, Newman-Keuls test in one-way ANOVA)

Comorbid chronic pain and mood disorder have been reported from animal models of neuropathic pain^11,13^ as well as patients with various chronic pain diseases^7,8^. To study whether the upregulation of the mGluR5 in the contributes to the comorbidity, we assessed the effect of the PrL-MPEP injection on the depressive-like behavior using forced swimming test (FST) paradigm. Consistent with previous reports^11,13^, neuropathic pain animals showed an increased immobility time in the FST chamber compared to sham control (Fig. 4A). We next tested the effect of MPEP injection into the bilateral PrL using separate groups of model animals. We found that the single-time PrL-MPEP treatment evokes rapid antidepressant effect on neuropathic pain animals. MPEP-treated SNL rats showed significantly reduced immobility time in FST compared to vehicle-treated SNL rats. Consequently, the immobility time of MPEP-treated SNL rats returned to the control level (Fig. 4A). Notably, the PrL-MPEP treatment was effective only in SNL animals, as the immobility time of MPEP-treated sham group rats was comparable with that of the vehicle-treated sham group. Regarding the upregulation of mGluR5 shown from PET imaging analysis, these data suggests that the increased mGluR5 action in the caudal PrL plays an important role in the pain-induced depressive-like behavior. To rule out the possibility that the reduction of immobility time of SNL rats in the FST was mediated by an effect on the basal activeness of the animals rather than anti-depressive effect, we measured the activity level of the rats in the open field test. Indeed, the PrL-MPEP treatment could not alter the activity level of the animals in the open field in both SNL and sham groups (Fig. 4B). Interestingly, the PrL-MPEP treatment significantly increased center zone visiting duration only in SNL group animals, suggesting its relieving effect from unpleasantness also leads to induction of a powerful anxiolytic effect (Fig. 4C).

### The antagonism of the mGluR5 in the PrL attenuates the aversive state of neuropathic pain condition

**Figure 5:**
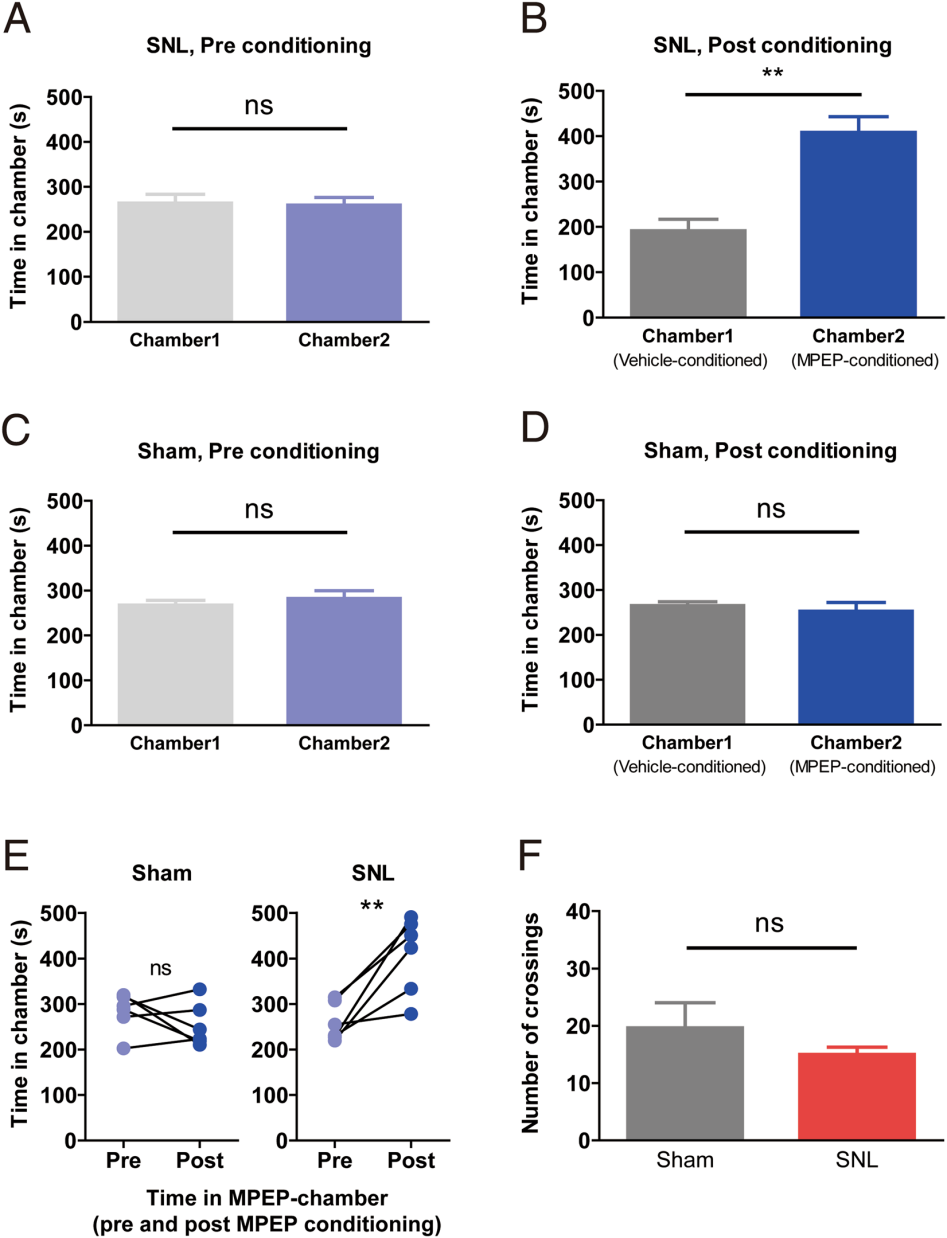
Neuropathic pain animals show preference to the PrL-MPEP treatment. (**A**) The absence of chamber preference before the conditioning, SNL group. (**B**) SNL group animals showed preference to the MPEP-conditioned chamber (n = 6, **p = 0.0043, Mann-Whitney test. (**C**) The absence of chamber preference before the conditioning, sham grou. (**D**) The preference to MPEP-conditioned chamber was not produced in sham group animals (n = 6). (**E**) The difference of the time spent in the MPEP-chamber before and after the conditioning. The time spent in the MPEP-conditioned chamber was significantly increased only in SNL group animals (**p = 0.007, paired t-test between pre- and post-conditioning data. (**F**) The number of crossings to the other chamber was not different between groups during the post-conditioning test period.

The mPFC has previously been shown to play an important role in the goal-directed behavior via processing of motivation coding and decision making. Thus, one might argue that the effects of the PrL-MPEP on the withdrawal behavior and immobility behavior we observed above might be caused by disturbance of these executive functions rather than intervention to the affective perception of the aversive state. To verify this issue, we assessed the effect of the PrL-MPEP on the formation of preference memory using conditional place preference (CPP) test. Before drug conditioning process of SNL animals, time spent in each chamber was identical between each chamber (Fig. 5A). After conditioning process, SNL animals spent significantly more time in the chamber in which they were conditioned with MPEP treatment into the PrL (Fig. 5B). As the place preference is formed by reinforcement memory related to the treatment, this result indicates that the SNL animals perceived the PrL-MPEP treatment as a reward (relief from the aversive state). In contrast, animals of sham surgery group showed no preference to the chamber conditioned with the PrL-MPEP treatment (Fig. 5C and D). We interpret that the animals without chronic pain perceived the PrL-MPEP treatment as neither reward nor punishment, as the time spent in each chamber was not altered compared to the pre-conditioning level in the sham group. The chamber preference appeared only in SNL group (Fig. 5E). A number of midline crossings to other chambers was not significantly different between groups during the test period (Fig. 5F), which confirms that the effect of the intervention on the basal activeness of the animals was not different between groups^31^. These results imply that the chamber preference formed by the PrL-MPEP in SNL group is caused by the relief from pain and/or pain-induced depression, and supports that the PrL-MPEP effect on the withdrawal behavior and immobility time is also mediated by the reduction of unpleasant affection. Taken together, these data demonstrate the effect of the mGluR5 blockade in the PrL on relieving the aversive state of the chronic pain condition.

### mGluR5 overexpression in the PrL induces mechanical allodynia, depressive-like behavior, and anxiety-like behavior

**Figure 6:**
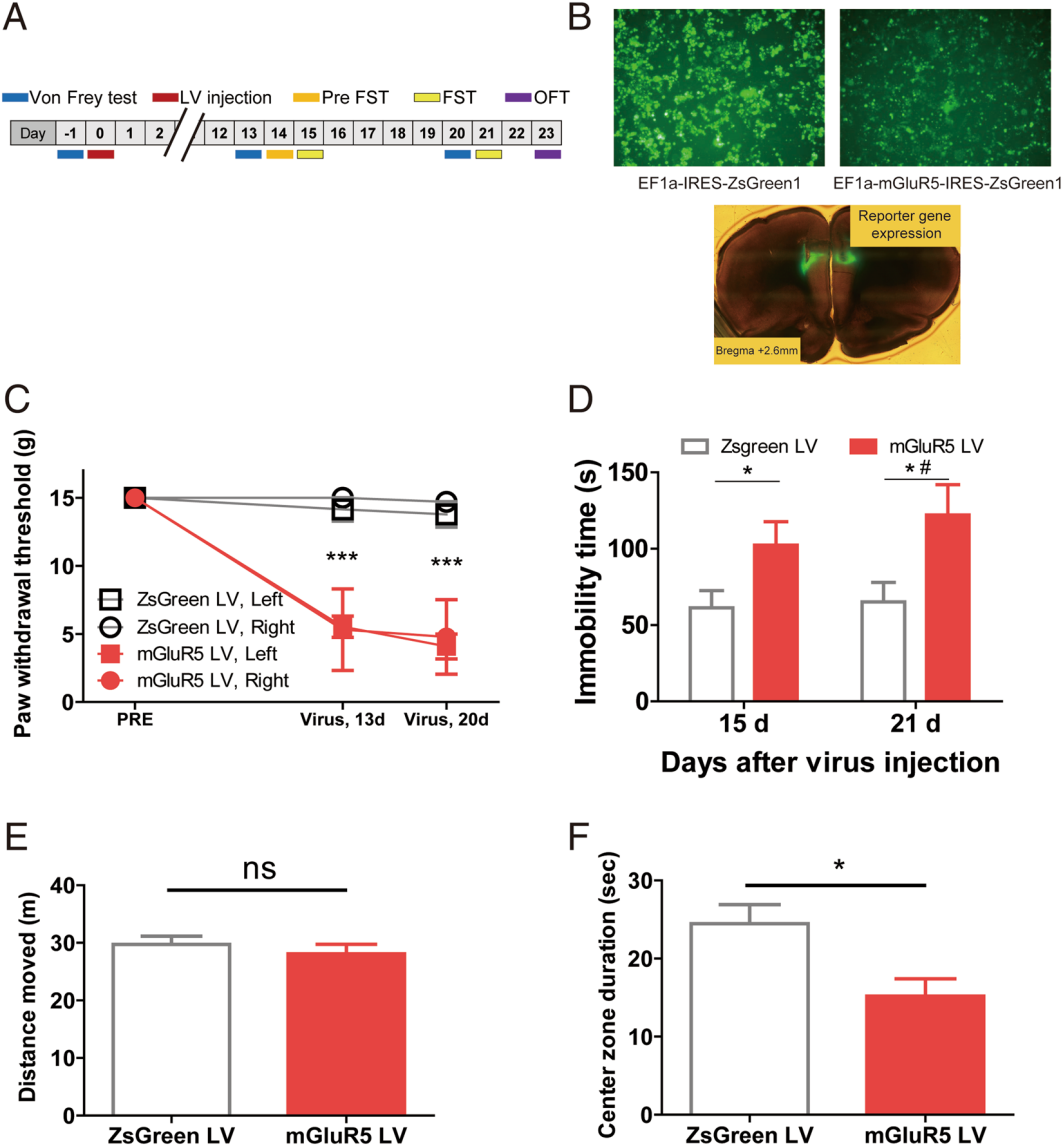
Lentiviral overexpression of mGluR5 in the mPFC induces comorbid pain and mood disorder. (**A**) Experimental design of lentiviral overexpression of mGluR5 and behavioral tests. (**B**) Confirmation of reporter gene expression *in vitro* (HEK293 cells) and *in vivo* (the PrL). (**C**) The injection of mGluR5 LV into the mPFC of naïve rats resulted in a reduction of the hindpaw withdrawal threshold (n = 10 per each group, *** p < 0.001, Bonferroni test in two-way repeated measures ANOVA). Consequently, tactile hypersensitivity comparable to SNL-induced mechanical allodynia was manifested. (**D**) The animals with mGluR5 LV injection showed higher immobility time in the FST (n = 10 per each group, *p < 0.05 with two sample t-test, #p < 0.05 with Bonferroni test in two-way repeated measures ANOVA). The higher level of this despair behavior implies that the animals with mGluR5 LV treatment were more vulnerable to depression compared to control. (**E**) Total distance traveled in the OFT was not altered by mGluR5 overexpression (n = 10 per each group), which confirms that the basal activeness was not affected. (**F**) The center zone duration in the OFT was decreased by the mGluR5 overexpression (n = 10 per each group, *p = 0.0118 with two sample t-test), which could be interpreted as increased anxiety level.

We asked whether the upregulation of mGluR5 in the PrL is sufficient to cause the both of pain behavior and depressive-like behavior. To confirm the causal relationship between the molecular change and the behavior, we injected lentivirus expressing mGluR5 (mGluR5 LV) into the bilateral PrL of naïve animals and observed the behavioral change (Fig. 6A and B). Lentivirus expressing ZsGreen1 (ZsGreen LV) was used as a control. In the behavior experiments, we observed that the rats subjected to the lentiviral overexpression of mGluR5 in the PrL showed reduced paw withdrawal threshold in the von Frey test (Fig. 6C) even though the SNL surgery was absent in these animals. In consequence, tactile hypersensitivity comparable to nerve injury-induced mechanical allodynia was manifested. Furthermore, subjects developed depressive-like behavior shown by increased immobility time in the FST (Fig. 6D). Basal activity level in the open field was not altered (Fig. 6E). In addition to the higher level of the despair behavior, animals showed the decreased center-zone duration (Fig. 6F) in the open field test. This implicates that the anxiety-like state was also developed in the subjects. These data demonstrate that the overexpression of mGluR5 in the PrL altered the behavioral coping strategy to aversive events without alteration of basal activeness, and implies that this treatment made animals more vulnerable to depression and anxiety.

These findings indicate the elevated mGluR5 in the PrL induce both pain and mood disorders, showing the causal relationship between upregulation of the PrL-mGluR5 in the chronic pain state and behavioral alterations.

## Discussion

As a modulator of the glutamatergic transmission, the mGluR5 is involved in the neuronal alteration observed from various neurological disorders^32^. The mGluR5 not only mediates plastic change of the nervous system but also shows expressional plasticity of itself in physiological and pathological conditions. In this study, we found that the mGluR5 level in the brain shows a plastic change in chronic pain condition and this alteration is a crucial mechanism of pain facilitation and negative mood behaviors.

We identified brain regions with the altered mGluR5 level in chronic neuropathic pain state and sought for specific brain region of which the alteration is responsible for comorbid chronic pain and depression. Our findings provide evidence which shows that the upregulation of mGluR5 level in the mPFC region is critical for the chronic neuropathic pain. We further showed that the upregulated mPFC-mGluR5 also plays a significant role in the manifestation of depressive-like behavior as well as pain behavior. Indeed, pharmacological blockade of mPFC-mGluR5 in our chronic neuropathic pain model animal was sufficient to relieve the both of the nerve injury-induced pain and the depression symptoms which are experimentally assessed by paw withdrawal threshold in von Frey test and immobility time in FST respectively. Conversely, lentiviral overexpression of mGluR5 in the mPFC of naïve animals resulted in the manifestation of tactile hypersensitivity and depressive-like behavior, further supporting the causal role of the increased mGluR5 in the mPFC.

A significant role of the mPFC has been implicated from both research fields of pain and depression performed with human and animal subjects. The rodent mPFC consists of subregions of ACC, PrL, and IL and each subregion show distinct actions on the pain as well as mood symptoms. The PrL was mainly focused on the pharmacological experiments of the current study, as we found that the mPFC cluster with a higher level of mGluR5 mainly consisted of caudal part PrL. The neurons located in the region are known to project to pain-related brain areas including ACC, insular cortex, periaqueductal gray, caudate putamen, amygdala, somatosensory cortex, thalamus and nucleus accumbens^33–37^. Interestingly, several target brain areas overlap with the brain regions in which the mGluR5 level was significantly altered in pain state in our PET analyses. Regarding the known role of these brain regions in the multiple aspects of pain and relevant emotional affection, the pain-induced alteration of the mGluR5 level in these regions would be also involved in the processing pathological and/or physiological aversiveness.

Although we focused on the higher level of mGluR5 level in the PrL subregion of the mPFC in the current study, mGluR5 in other brain regions should be the target of further studies to elucidate the mechanisms of encoding aversion and depression. Of interest, the lower level of mGluR5 was shown in the brain regions of insular cortex and nucleus accumbens of neuropathic pain group in our PET analyses. Given that these brain areas are involved in the altered sensory processing, abnormal perception related to unpleasantness, and decreased motivation in chronic pain state^38–43^, down-regulation of the mGluR5 in these areas would play a part in pain chronification and following negative affection. We produced the brain map of altered mGluR5 in the pathological pain condition, providing the basis for studying the actions of the neural circuits on the pain and negative emotion.

Whether the mGluR5s are altered in a specific subpopulation of mPFC neurons in pain state remains unknown in our study. Previous studies suggested that the decreased activity of pyramidal neurons in the PrL plays an essential role in pain and pain-induced affective symptoms^30,44^, and increased GABAergic inhibition is responsible for the reduction of pyramidal neuronal activity^31^. Although activation of mGluR5 generally promotes neuronal excitability, these previous studies raise the possibility that the upregulation of mGluR5 observed in our PET data might enhance inhibitory effects to the pyramidal neurons. This can be achieved by (1) upregulation of mGluR5 in the GABAergic inhibitory neurons. A recent study performed in non-pain animal showed that the systemic injection of mGluR5 antagonist induces the anti-depressive effect in naïve mice, and this effect is mediated by mGluR5 expressed in parvalbumin-expressing GABAergic interneurons in the mPFC^29^. Alternatively, (2) the mGluR5 might be upregulated in the glutamatergic neurons in the mPFC which drive excitation of GABAergic neurons. This view is supported by a recent study which showed that the increased GABAergic activity in the PrL of neuropathic pain animal critically depends on the enhanced excitatory synaptic input onto the GABAergic neurons^31^. Thus, upregulation of the mGluR5 might occur in the specific glutamatergic neurons in the mPFC area, which leads to facilitation of feed-forward inhibition to the pyramidal neurons via increased glutamate release to the GABAergic neurons. By dissecting the subpopulation of neurons associated with upregulated mGluR5 in the further study, more detailed mechanisms and novel therapies could be discovered.

Currently, both chronic pain diseases and depressive disorders lack methods for objective evaluation in the clinic, and the techniques to investigate mGluR5 level in the brain is considered to be a promising approach for the assessment of the pain and/or depression in clinical trials. In the case of non-pain depression, however, there is an inconsistency in the previous studies regarding the alteration of brain mGluR5^45–49^. A previous study has reported that the female patients with major depressive disorder showed the higher levels of mGluR5 gene expression in the PFC subregion whereas male patients showed the lower levels of mGluR5^48^. Interestingly, a previous animal study has reported that male rats but not female rats show higher levels of prefrontal mGluR5 in the depressive state induced by prenatal chronic mild stress^50^. Other studies have reported that male mice with mGluR5 knockout showed increased depressive-like behavior, indicating that the activity of the mGluR5 primarily facilitates the depression^29,51^. In that, the inconsistency shown from human studies is likely due to differences in gender^48^, age, onset time, pathophysiological differences of the subjects^47^, and a specific subpopulation of the neurons in which mGluR5 is altered^29^. As the depression is the complex mental problem, there might be discrepancies as well as common mechanisms between non-pain depressive disorders and depression following pain. Thus, whether suffering negative mood with the different origins for a long period in the certain circumstances eventually upregulate or downregulate prefrontal mGluR5 might be case-specific. In this regards, further studies are necessary for the diagnostic application of the prefrontal mGluR5 measurement to human subjects. A translational step is also required regarding the debate over the homology between regions in rodent and primate prefrontal cortex.

The anti-depressive effect of mGluR5 antagonists, in contrast, has been consistently reported from both of pre-clinical and clinical studies^29,52–60^. As such, the PrL-MPEP treatment in our study showed a rapid anti-depressive effect comparable to fast-acting antidepressant drugs, as the treatment reduced immobility time in the FST which was performed at 30 minutes after single-time injection. In addition, the current study supports that the mPFC-mGluR5 could be an attractive therapeutic target for the chronic pain as well as depressive disorder. In our von Frey test, the effect of consecutive PrL-MPEP treatment on the paw withdrawal threshold had not fully disappeared after 24 hours, suggesting the possible long-term plastic change. These characteristics make the mPFC-mGluR5 an attractive target of intervention which might overcome the limit of current therapeutic approaches.

## Materials and Methods

### Animals

Adult male Sprague-Dawley rats (Samtako, Seoul, Korea) were housed two per cage at a constant temperature of 23 ± 1 °C under a 12 h light/dark cycle. Food and water were available ad libitum. All the experimental procedures were approved by Institutional Animal Care and Use Committee at Seoul National University and were performed according to the Ethical Guidelines of the International Association for the Study of Pain.

### Spinal Nerve Ligation (SNL)

Adult rats (8 weeks old) were subjected to SNL or sham surgery^23^. Isofluorane was inhaled from 15 mins before surgery till the end. In SNL surgery, the right L5 spinal nerve was ligated after elimination of transverse process and fascia, to induce chronic neuropathic pain. In sham surgery, the L5 spinal nerve was kept intact after L5 transverse process elimination. Animals were moved to their cages immediately after surgery process and monitored during recovery. Rats with foot-drop were excluded from the further experiments.

### Paw withdrawal Thresholds

Paw withdrawal thresholds of the affected area in hind paw were measured at one day before and 1, 5, 9, 15 days after surgery using von Frey filaments. Dixon’s up-down method and Chaplan’s calculation method were used^61,62^ and withdrawal threshold of 15 g was applied as the cut-off. Animals with a sensitive hind paw (withdrawal threshold less than 10 g) at pre-surgery condition were not subjected to surgery. There were animals without mechanical hypersensitivity at 5 days after SNL surgery (less than 15% total) and these animals were excluded from the further study.

### PET imaging design

PET images were acquired from the SNL group and sham group rats, between the 16^th^ and the 25^th^ day after the surgery. Animals were anesthetized and maintained with 1.5% isoflurane and received a tail vein injection of [11C] ABP688 (5.05–16.15 MBq/100 g). Brain scans were processed by micro-PET/CT scanner (eXplore VISTA, GE Healthcare) with list-mode for 60 minutes. After finishing scan procedure, list mode data were reconstructed into a single static image of the full 60 minutes and into dynamic frames of 6 * 30 sec, 7 * 60 sec, and 5 * 600 sec duration using 3-dimensional ordered-subsets expectation maximum (OSEM) algorithm with scatter correction and random correction. Voxel size was 0.3875 * 0.3875 * 0.775 mm. Each image was reconstructed in proportion to standardized uptake value (SUV).

### Image preprocessing

Static images of the full 60 minutes from each dataset were coregistered with a standard rat MRI template^63^ and the calculated transformation parameters were applied to the respective binned images. Coregistration was visually confirmed and then the time-activity curves (TAC) for the cerebellum were extracted from the series of binned images. The non-displaceable binding potential (BP_ND_) of [11C]ABP688 was calculated by use of the simplified reference tissue model with the cerebellum as a reference region. Images were cropped and averaged to make ABP688-PET brain template and then all the images were spatially normalized to this ABP688-PET brain template. Voxels were resampled to 0.2 * 0.2 * 0.2 mm and smoothed with a Gaussian filter of 0.8 mm full-width at half maximum. Images were processed using Statistical Parametric Mapping 8 software package (SPM8), MarsBaR toolbox and imgsrtm program of Turku PET Centre.

### SPM analysis

A voxel-by-voxel comparison of mGluR5 availability between the two groups was performed using the SPM8 software. A two-sample t-test was used to assess differences between SNL group and sham control. An uncorrected p-value threshold of 0.005 and an extent threshold of 20 voxels were used to determine statistical significance. The statistical map was overlaid to MRI template image for visualization purpose.

### Cannula implantation and drug injection

On the 9^th^ day after SNL or sham surgery, rats were deeply anesthetized and guide cannulas were implanted in the PrL bilaterally. The coordinates of AP + 2.6 mm, ML ± 0.8 mm, DV −2.9 mm from the bregma were targeted, which made the tip of the guide cannula locate 0.7 mm above the injection site. After the guide cannula was settled by dental cement, rats were returned to their home cage and at least 7 days of recovery period was given. After the recovery period, the rats were handled once a day for 3 consecutive days prior to drug injection. Microinjection of drug solution into the target region was conducted using internal cannula which has a length of 0.7 mm additional projection beyond the tip of the guide cannula (final target DV −3.6 mm). In the case of the dorsal and ventral parts, targeted coordinates were identical except final target DV −2.0 mm and −5.4 mm.

A volume of 0.5 ul drug solutions (25 nmol of MPEP) was injected to each side of mPFC and behavioral experiments were followed. In the case of MTEP solution, 10 nmol of MTEP in the same volume was used. Behavioral experiments were performed on a separate group of animals with a single drug application unless specified otherwise. After finishing the experiments, each animal was deeply anesthetized and injected with a methyl green dye to mark the injection sites of the brain. Each animal went under cardiac perfusion of phosphate-buffered saline and 4% paraformaldehyde. Brains were extracted, stored in formalin and sliced in coronal sections. Only data from rats with confirmed placements of the cannulas were used.

### Forced swimming test (FST)

On the 22^nd^ day after SNL or sham surgery, the rat was placed in a swim cylinder (20 cm in diameter and 45 cm height) filled with water at 23 °C (depth of 30 cm) and underwent a pre-test swimming of 15 minutes. After the pre-test swimming, the rat was dried with the towels and returned to its home-cage. On the next day (the 23^rd^ day after the surgery), one of the solutions (DMSO vehicle or MPEP solution) was randomly selected and injected into the bilateral PrL of the rat via implanted cannula. At 30 minutes after the injection, the rat was placed in the swim cylinder for a 5 minutes swimming^64^. In the case of the experiment with lentiviral expression, 15 minutes of pre-test swimming was performed on the 14^th^ day after the injection of mGluR5-LV or ZsGreen-LV into the bilateral PrL of the naïve rat. Repeated 5 minutes of swimming tests were performed on the 15^th^ and 21^st^ day after the LV injection^65^. All the tests were video-recorded and the immobility time was analyzed from 5 minutes swimming. Immobility was defined as when rat had no activity except for those necessary to keep its head above water.

### Open field test (OFT)

The rat was placed in the center of an open field box (60 cm * 60 cm square, 30 cm height) under low-light illumination (<20 lux) and was video-recorded during 5 minutes. The experimenter was blinded to the group and total distance moved and time spent in the center zone during the test were analyzed automatically using EthoVision XT software (Noldus). Center zone was defined as a zone of 30 cm * 30 cm center square. In the case of the MPEP experiment, the drug was injected into the medial prefrontal cortex (mPFC) via implanted cannula and the rat was returned to its home-cage. OFT was performed 30 minutes after the drug injection.

### Conditioned place preference test (CPP)

Cannulated rats had a 3 days protocol for 3 chamber CPP test^66^. The CPP chamber consisted of two pairing chambers (vertical versus horizontal visual cues) connected by a neutral chamber. All animals were exposed to the experimental room environment three days prior to the beginning of the main experiment for adaptation purpose. On the first day of the main experiment, the animal was allowed to access to all chambers freely for 15 minutes. The time spent in each chamber was analyzed to confirm the absence of pre-conditioning chamber preference. Animals spending less than 20% of the time in any one chamber at this stage were excluded from further experiments. On the second day, the vehicle DMSO solution was injected into the bilateral PrL, and the animal was returned to its home-cage. The 30 minutes of the waiting period was given. Then the animal was placed in a randomly assigned chamber (with vertical or horizontal visual cue) for 30 minutes. After 4 hours, the MPEP solution was injected to the bilateral PrL of the same animal and 30 minutes of home-cage waiting was followed. And then the animal was placed in the counterbalanced chamber for 30 minutes. The animal was not allowed to access to the other chambers during these pairing periods. On the third day, the animal was placed into the neutral chamber and was allowed to freely access to all chambers for the 15 minutes. The time spent in each chamber was calculated and compared.

### Lentiviral overexpression of mGluR5

The viral vector was a generous gift from Professor Chul Hoon Kim, and lentivirus expressing mGluR5 or ZsGreen1 was produced and verified as previously described^67^. In brief, the cDNA of mGluR5 (primer forward: ATGGTCCTTCTGTTGATTCTGTCAG, reverse: TCACAACGATGAAGAACTCTGCG) was amplified and inserted into the lentiviral vector pLVX-EF1α–IRES-ZsGreen1 (Clontech, Catalog No. 631982), and the plasmid was transfected into HEK293FT cells of 60–70% confluency together with psPAX2 (Addgene), pMD2G (Addgene) and polyethyleneimine solution (sigma-Aldrich). Cell media were harvested, centrifuged and then ultracentrifuged. After the re-suspension, the virus was aliquoted and stored at −80 °C until use. The virus was validated with Western blot analysis in virus-treated HEK293FT cells using mGluR5 antibody (Abcam, EPR2425Y).

The virus expressing EF1α-IRES-Zsgreen1 (ZsGreen LV, titer = 3*10^8 TU/ml) or EF1α-mGluR5-IRES-Zsgreen1 (mGluR5 LV, titer = 1*10^8 TU/ml) was injected to the bilateral mPFC (coordinates of AP + 2.6 mm, ML ± 0.8 mm, DV −3.6 mm from the bregma) of naïve rats. Behavioral experiments were started 13 days after the virus injection. After finishing the experiments, the brains were extracted from each animal and coronal sections containing the mPFC region were prepared. The correct injection sites were confirmed in all animals. In the brain slices, precise delivery of the virus was confirmed by visualizing ZsGreen1-expressing neurons in the mPFC region.

### Drugs

2-Methyl-6-(phenylethynyl)pyridine hydrochloride (MPEP) and 3-((2-Methyl-1,3-thiazol-4-yl)ethynyl)pyridine hydrochloride (MTEP) were purchased from Tocris. Drugs were dissolved in DMSO in 100 mM stock solutions and diluted to final concentration with distilled water when used.

### Statistical analysis

Paw withdrawal thresholds were analyzed using two-way repeated measures ANOVA with time serving as a within-subject factor. For FST and OFT, group data were analyzed using a one-way ANOVA followed by post hoc test as specified. For CPP experiments, the time difference between pre- and post-conditioning was compared using paired t-test in each group, and the preference to the MPEP-conditioned chamber (CPP index) was compared between groups using Mann-Whitney test.

### Data Availability

The datasets generated during and/or analyzed during the current study are available from the corresponding author on reasonable request.

## References

[1] Zhuo M (2008). Cortical excitation and chronic pain. Trends Neurosci..

[2] Metz AE, Yau H-J, Centeno MV, Apkarian AV, Martina M (2009). Morphological and functional reorganization of rat medial prefrontal cortex in neuropathic pain. Proc. Natl. Acad. Sci. USA.

[3] Seifert F, Maihöfner C (2009). Central mechanisms of experimental and chronic neuropathic pain: Findings from functional imaging studies. Cell. Mol. Life Sci..

[4] Maihöfner C, Forster C, Birklein F, Neundörfer B, Handwerker HO (2005). Brain processing during mechanical hyperalgesia in complex regional pain syndrome: A functional MRI study. Pain.

[5] Seminowicz DA (2009). MRI structural brain changes associated with sensory and emotional function in a rat model of long-term neuropathic pain. Neuroimage.

[6] Thompson SJ (2014). NeuroImage Metabolic brain activity suggestive of persistent pain in a rat model of neuropathic pain. Neuroimage.

[7] Radat F, Margot-Duclot A, Attal N (2013). Psychiatric co-morbidities in patients with chronic peripheral neuropathic pain: a multicentre cohort study. Eur. J. Pain.

[8] Gupta A (2007). The role of psychosocial factors in predicting the onset of chronic widespread pain: Results from a prospective population-based study. Rheumatology.

[9] Turk DC, Audette J, Levy RM, Mackey SC, Stanos S (2010). Assessment and treatment of psychosocial comorbidities in patients with neuropathic pain. Mayo Clin. Proc..

[10] Shi M, Qi WJ, Gao G, Wang JY, Luo F (2010). Increased thermal and mechanical nociceptive thresholds in rats with depressive-like behaviors. Brain Res..

[11] Li JX (2015). Pain and depression comorbidity: A preclinical perspective. Behav. Brain Res..

[12] Suzuki T (2007). Experimental neuropathy in mice is associated with delayed behavioral changes related to anxiety and depression. Anesth. Analg..

[13] Leite-Almeida H, Pinto-Ribeiro F, Almeida A (2015). Animal models for the study of comorbid pain and psychiatric disorders. Mod. Trends Pharmacopsychiatry.

[14] Kim SK (2016). Cortical astrocytes rewire somatosensory cortical circuits for peripheral neuropathic pain. J. Clin. Invest..

[15] Matos SC, Zhang Z, Seguela P (2015). Peripheral Neuropathy Induces HCN Channel Dysfunction in Pyramidal Neurons of the Medial Prefrontal Cortex. J. Neurosci..

[16] Kolber, B. J. MGluRs head to toe in pain. Progress in Molecular Biology and Translational Science **131**, (Elsevier Inc., 2015). 10.1016/bs.pmbts.2014.12.003 25744677

[17] Marsden WN (2013). Synaptic plasticity in depression: Molecular, cellular and functional correlates. Progress in Neuro-Psychopharmacology and Biological Psychiatry.

[18] Duman RS, Aghajanian GK, Sanacora G, Krystal JH (2016). Synaptic plasticity and depression: new insights from stress and rapid-acting antidepressants. Nat. Med..

[19] Bannerman DM (2014). Hippocampal synaptic plasticity, spatial memory and anxiety. Nat. Rev. Neurosci..

[20] Bushnell MC, Čeko M, Low LA (2013). Cognitive and emotional control of pain and its disruption in chronic pain. Nat Rev Neurosci.

[21] Yalcin I, Barthas F, Barrot M (2014). Emotional consequences of neuropathic pain: Insight from preclinical studies. Neurosci. Biobehav. Rev..

[22] Baliki MN (2006). Chronic pain and the emotional brain: specific brain activity associated with spontaneous fluctuations of intensity of chronic back pain. J. Neurosci..

[23] Ho Kim S, Mo Chung J (1992). An experimental model for peripheral neuropathy produced by segmental spinal nerve ligation in the rat. Pain.

[24] Lemogne, C., Delaveau, P., Freton, M., Guionnet, S. & Fossati, P. Medial prefrontal cortex and the self in major depression. *Journal of Affective Disorders* **136**, (2012). 10.1016/j.jad.2010.11.034 21185083

[25] Treadway MT (2015). Illness progression, recent stress, and morphometry of hippocampal subfields and medial prefrontal cortex in major depression. Biol. Psychiatry.

[26] Alexander WH, Brown JW (2012). Medial Prefrontal Cortex as an action-outcome predictor. Nat. Neurosci..

[27] McEwen AM (2012). Increased Glutamate Levels in the Medial Prefrontal Cortex in Patients with Postpartum Depression. Neuropsychopharmacology.

[28] Etkin A, Egner T, Kalisch R (2011). Emotional processing in anterior cingulate and medial prefrontal cortex. Trends in Cognitive Sciences.

[29] Lee K-W (2015). Alteration by p11 of mGluR5 localization regulates depression-like behaviors. Mol. Psychiatry.

[30] Wang G-Q (2015). Deactivation of excitatory neurons in the prelimbic cortex via Cdk5 promotes pain sensation and anxiety. Nat. Commun..

[31] Zhang Z (2015). Role of Prelimbic GABAergic Circuits in Sensory and Emotional Aspects of Neuropathic Pain. Cell Rep..

[32] Bordi, F. & Ugolini, A. Group I Metabotropic Glutamate Receptors: Implications for Brain Diseases. **59** (1999). 10.1016/s0301-0082(98)00095-1 10416961

[33] Gorelova N, Yang CR (1996). The course of neural projection from the prefrontal cortex to the nucleus accumbens in the rat. Neuroscience.

[34] Vertes RP (2004). Differential Projections of the Infralimbic and Prelimbic Cortex in the Rat. Synapse.

[35] Cheriyan, J., Kaushik, M. K., Ferreira, A. N. & Sheets, P. L. Specific targeting of the basolateral amygdala to projectionally defined pyramidal neurons in prelimbic and infralimbic cortex. *eNeuro* **3** (2016). 10.1523/ENEURO.0002-16.2016 PMC4804386 27022632

[36] Sesack SR, Deutch AY, Roth RH, Bunney BS (1989). Topographical organization of the efferent projections of the medial prefrontal cortex in the rat: An anterograde tract-tracing study with Phaseolus vulgaris leucoagglutinin. J. Comp. Neurol..

[37] Hoppa MB (2015). Connectivity of mouse somatosensory and prefrontal cortex examined with trans-synaptic tracing. Nat. Neurosci..

[38] Schwartz, N. *et al*. Decreased motivation during chronic pain requires long-term depression in the nucleus accumbens. *Science* (80*−*) 345 (2014). 10.1126/science.1253994 PMC4219555 25082697

[39] Starr CJ (2009). Roles of the insular cortex in the modulation of pain: insights from brain lesions. J. Neurosci..

[40] Chang Pei-Ching, Pollema-Mays Sarah Lynn, Centeno Maria Virginia, Procissi Daniel, Contini Massimo, Baria Alex Tomas, Martina Marco, Apkarian Apkar Vania (2014). Role of nucleus accumbens in neuropathic pain: Linked multi-scale evidence in the rat transitioning to neuropathic pain. Pain.

[41] Schreckenberger M (2005). The unpleasantness of tonic pain is encoded by the insular cortex. Neurology.

[42] Kaneko H (2017). Dysfunction of Nucleus Accumbens Is Associated With Psychiatric Problems in Patients With Chronic Low Back Pain. Spine (Phila. Pa. 1976)..

[43] Benarroch EE (2016). Involvement of the nucleus accumbens and dopamine system in chronic pain. Neurology.

[44] Kelly CJ, Huang M, Meltzer H, Martina M (2016). Reduced Glutamatergic Currents and Dendritic Branching of Layer 5 Pyramidal Cells Contribute to Medial Prefrontal Cortex Deactivation in a Rat Model of Neuropathic Pain. Front. Cell. Neurosci..

[45] Esterlis I (2017). Ketamine-induced reduction in mGluR5 availability is associated with an antidepressant response: an [11C]ABP688 and PET imaging study in depression. Mol. Psychiatry.

[46] Deschwanden A (2011). Reduced metabotropic glutamate receptor 5 density in major depression determined by [11C]ABP688 PET and postmortem study. Am. J. Psychiatry.

[47] DeLorenzo C (2015). Characterization of brain mGluR5 binding in a pilot study of late-life major depressive disorder using positron emission tomography and [(1)(1)C]ABP688. Transl. Psychiatry.

[48] Gray AL, Hyde TM, Deep-Soboslay A, Kleinman JE, Sodhi MS (2015). Sex differences in glutamate receptor gene expression in major depression and suicide. Mol. Psychiatry.

[49] Abdallah CG (2017). Metabotropic Glutamate Receptor 5 and Glutamate Involvement in Major Depressive Disorder: A Multimodal Imaging Study. Biol. Psychiatry Cogn. Neurosci. Neuroimaging.

[50] Wang Y (2015). Prenatal chronic mild stress induces depression-like behavior and sex-specific changes in regional glutamate receptor expression patterns in adult rats. Neuroscience.

[51] Witkin JM, Marek GJ, Johnson BG, Schoepp DD (2007). Metabotropic glutamate receptors in the control of mood disorders. CNS Neurol Disord Drug Targets.

[52] Pilc A, Chaki S, Nowak G, Witkin JM (2008). Mood disorders: regulation by metabotropic glutamate receptors. Biochem. Pharmacol..

[53] Palucha A, Pilc A (2007). Metabotropic glutamate receptor ligands as possible anxiolytic and antidepressant drugs. Pharmacol. Ther..

[54] Hashimoto K, Malchow B, Falkai P, Schmitt A (2013). Glutamate modulators as potential therapeutic drugs in schizophrenia and affective disorders. Eur. Arch. Psychiatry Clin. Neurosci..

[55] Fuxe K, Borroto-Escuela DO (2015). Basimglurant for treatment of major depressive disorder: a novel negative allosteric modulator of metabotropic glutamate receptor 5. Expert Opin. Investig. Drugs.

[56] Kato T (2015). DSR-98776, a novel selective mGlu5 receptor negative allosteric modulator with potent antidepressant and antimanic activity. Eur. J. Pharmacol..

[57] Hughes ZA (2013). Negative allosteric modulation of metabotropic glutamate receptor 5 results in broad spectrum activity relevant to treatment resistant depression. Neuropharmacology.

[58] Chaki S, Ago Y, Palucha-Paniewiera A, Matrisciano F, Pilc A (2013). mGlu2/3 and mGlu5 receptors: Potential targets for novel antidepressants. Neuropharmacology.

[59] Lindemann L (2015). Pharmacology of Basimglurant (RO4917523, RG7090), a Unique Metabotropic Glutamate Receptor 5 Negative Allosteric Modulator in Clinical Development for Depression. J. Pharmacol. Exp. Ther..

[60] Park M, Niciu MJ, Zarate CA (2015). Novel Glutamatergic Treatments for Severe Mood Disorders. Curr. Behav. Neurosci. Reports.

[61] Dixon WJ (1965). The up-and-down method for small samples. J. Am. Stat. Assoc..

[62] Chaplan SR, Bach FW, Pogrel JW, Chung JM, Yaksh TL (1994). Quantitative assessment of tactile allodynia in the rat paw. J. Neurosci. Methods.

[63] Schweinhardt P, Fransson P, Olson L, Spenger C, Andersson JLR (2003). A template for spatial normalisation of MR images of the rat brain. J. Neurosci. Methods.

[64] Slattery D (2012). a & Cryan, J. F. Using the rat forced swim test to assess antidepressant-like activity in rodents. Nat. Protoc..

[65] Mezadri TJ, Batista GM, Portes AC, Marino-Neto J, Lino-de-Oliveira C (2011). Repeated rat-forced swim test: Reducing the number of animals to evaluate gradual effects of antidepressants. J. Neurosci. Methods.

[66] King T (2009). Unmasking the tonic-aversive state in neuropathic pain. Nat. Neurosci..

[67] Shin S (2015). mGluR5 in the nucleus accumbens is critical for promoting resilience to chronic stress. Nat. Neurosci..

