# Publisher Correction: Upregulation of prefrontal metabotropic glutamate receptor 5 mediates neuropathic pain and negative mood symptoms after spinal nerve injury in rats

**DOI:** 10.1038/s41598-018-19727-x

**Published:** 2018-02-06

**Authors:** Geehoon Chung, Chae Young Kim, Yeong-Chan Yun, Sang Ho Yoon, Myoung-Hwan Kim, Yu Kyeong Kim, Sang Jeong Kim

**Affiliations:** 1 Department of Physiology, Seoul National University College of Medicine, Seoul, Korea; 2 Department of Brain and Cognitive Sciences, Seoul National University College of Natural Sciences, Seoul, Korea; 3 Department of Biomedical Sciences, Seoul National University College of Medicine, Seoul, Korea; 4 Department of Nuclear Medicine, Seoul National University College of Medicine, Seoul, Korea; 5 Neuroscience Research Institute, Seoul National University College of Medicine, Seoul, Korea

Correction to: *Scientific Reports* 10.1038/s41598-017-09991-8, published online 29 August 2017

In this Article, the two parts of Table 1 are merged together. The correct Table 1 appears below.

**Table 1:** Brain regions with significant differences between sham and SNL rats (two-sample t-test, p < 0.005, K_e_ > 20). The peak voxel location is represented by the distance from the bregma (mm).

Brain region	K_e_	T	Z	Peak p	Location (mm)		
					ML	AP	DV
**Sham < SNL**							
Medial prefrontal cortex (Prelimbic and infralimbic cortices)	97	5.50	4.06	2.54E-06	0.2	2.6	−4.8
Primary somatosensory cortex (Dysgranular zone)	24	5.88	4.23	1.16E-05	−4.6	−0.2	−3.4
Primary, secondary somatosensory cortices and caudate putamen	37	4.42	3.52	0.0002	4.8	−0.4	−5.2
Retrosplenial cortex	25	6.70	4.56	2.45E-05	0.2	−4.6	−2.4
Medial septum	30	3.51	2.98	0.0014	0.4	−0.8	−4.8
**Sham > SNL**							
Insular cortex and piriform cortex	102	7.64	4.89	4.98E-07	−3.2	3.6	−7.0
		4.73	3.69	0.0001	−3.4	3.0	−7.6
		4.60	3.62	0.0001	−3.6	2.6	−5.8
Insular cortex and primary somatosensory cortex	31	4.12	3.35	0.0004	−4.8	3.4	−4.8
Endopiriform nucleus	20	4.06	3.32	0.0005	3.8	1.4	−7.2
Nucleus accumbens	30	3.74	3.12	0.0009	1.6	1.8	−6.4
Piriform cortex	61	3.72	3.11	0.0009	−6.2	−4.8	−8.6
Corpus callosum and caudate putamen	36	3.71	3.11	0.0009	1.6	−0.4	−3.4
Corpus callosum and caudate putamen	76	3.65	3.07	0.0011	3.6	−2.0	−3.8

