# Publisher Correction: Upregulation of prefrontal metabotropic glutamate receptor 5 mediates neuropathic pain and negative mood symptoms after spinal nerve injury in rats

**DOI:** 10.1038/s41598-018-26993-2

**Published:** 2018-06-07

**Authors:** Geehoon Chung, Chae Young Kim, Yeong-Chan Yun, Sang Ho Yoon, Myoung-Hwan Kim, Yu Kyeong Kim, Sang Jeong Kim

**Affiliations:** 1 Department of Physiology, Seoul National University College of Medicine, Seoul, Korea; 2 Department of Brain and Cognitive Sciences, Seoul National University College of Natural Sciences, Seoul, Korea; 3 Department of Biomedical Sciences, Seoul National University College of Medicine, Seoul, Korea; 4 Department of Nuclear Medicine, Seoul National University College of Medicine, Seoul, Korea; 5 Neuroscience Research Institute, Seoul National University College of Medicine, Seoul, Korea

Correction to: *Scientific Reports* 10.1038/s41598-017-09991-8, published online 29 August 2017

In the original version of this Article, the authors Chae Young Kim, Sang Ho Yoon, Yu Kyeong Kim and Sang Jeong Kim were incorrectly indexed.

Additionally, the original version of this Article omitted an affiliation for Sang Jeong Kim. The correct affiliations are listed below:

Department of Physiology, Seoul National University College of Medicine, Seoul, Korea

Department of Brain and Cognitive Sciences, Seoul National University College of Natural Sciences, Seoul, Korea

Department of Biomedical Sciences, Seoul National University College of Medicine, Seoul, Korea

Neuroscience Research Institute, Seoul National University College of Medicine, Seoul, Korea

These errors have now been corrected in the HTML and PDF versions of this Article.

